# Biomedical titanium alloys with Young’s moduli close to that of cortical bone

**DOI:** 10.1093/rb/rbw016

**Published:** 2016-03-08

**Authors:** Mitsuo Niinomi, Yi Liu, Masaki Nakai, Huihong Liu, Hua Li

**Affiliations:** ^1^Institute for Materials Research, Tohoku University, 2-1-1, Katahira, Aoba-Ku, Sendai 980-8577, Japan and; ^2^Ningbo Institute of Materials Technology and Engineering, Chinese Academy of Sciences, Ningbo 315201, China

**Keywords:** titanium alloys, mechanical strength, Young’s modulus, TNTZ, biological performances, surface modification

## Abstract

Biomedical titanium alloys with Young’s moduli close to that of cortical bone, i.e., low Young’s modulus titanium alloys, are receiving extensive attentions because of their potential in preventing stress shielding, which usually leads to bone resorption and poor bone remodeling, when implants made of their alloys are used. They are generally β-type titanium alloys composed of non-toxic and allergy-free elements such as Ti–29Nb–13Ta–4.6Zr referred to as TNTZ, which is highly expected to be used as a biomaterial for implants replacing failed hard tissue. Furthermore, to satisfy the demands from both patients and surgeons, i.e., a low Young’s modulus of the whole implant and a high Young’s modulus of the deformed part of implant, titanium alloys with changeable Young’s modulus, which are also β-type titanium alloys, for instance Ti–12Cr, have been developed. In this review article, by focusing on TNTZ and Ti–12Cr, the biological and mechanical properties of the titanium alloys with low Young’s modulus and changeable Young’s modulus are described. In addition, the titanium alloys with shape memory and superelastic properties were briefly addressed. Surface modifications for tailoring the biological and anti-wear/corrosion performances of the alloys have also been briefly introduced.

## Introduction

With the rapid increase in the elderly population, disturbance of motility, which is one of the three main diseases: alongside cancer, circulatory diseases (cardiovascular diseases), is increasing among aged persons. The disturbance of motility is mainly caused by disordering of hard-tissue, i.e., cortical bone (hereafter bone). In such cases, the disordered bones are reconstructed by them with artificial implant devices such as bone plates, hip joints, spinal fixation devices and dental roots to maintain quality of life. A high load is usually applied on these implant devices. Therefore, metallic biomaterials are suitable candidates for constructing these implant devices. These biomaterials require high mechanical reliability and high corrosion resistance to prevent dissolution of metallic elements, and they should be composed of elements exhibiting low toxicity and allergic problems. Simultaneously, metallic biomaterials require a low Young’s modulus, close to that of bone (10–30 GPa), to prevent bone resorption and achieve good bone remodeling.

The biocompatibility of titanium and its alloys is superior to those of other representative metallic biomaterials such as stainless steel (especially SUS 316 L stainless steel) and Co–Cr–Mo alloys. The Young’s moduli of titanium and its alloys are smaller than those of SUS 316 L stainless steel and Co–Cr–Mo alloys, and other characteristics such as their corrosion resistance are also excellent. Furthermore, the balance between strength and ductility of titanium alloys is superior to those of other metallic materials. Therefore, titanium alloys have been receiving much attention for use in implant devices. For example, the most widely used Ti alloy, (α + β)-type Ti64 ELI (referred to as Ti64 ELI), for biomedical applications has a Young’s modulus (∼110 GPa) that is only about half of those of SUS 316 L stainless steel (∼200 GPa [1, 2]) and Co–Cr–Mo alloys (∼210 GPa [3]), which are still commonly used in implant devices. However, because of the toxicity of vanadium and the higher Young’s modulus of Ti64 ELI than that of the bone (∼10–30 GPa) [1, 3, 4], this alloy cannot be widely used in spinal fixture devices. Over the past two decades, many novel types of Ti alloys with good biocompatibility and a low Young’s modulus (∼60 GPa) similar to that of the bone have been developed for biomedical applications. For example, β-type Ti–29Nb–13Ta–4.6Zr (TNTZ) [5–8] has good mechanical properties, corrosion resistance and biocompatibility and a low Young’s modulus of ∼60 GPa. Therefore, TNTZ is considered to be a promising candidate for use as a next-generation metallic biomaterial.

Unfortunately, none of the aforementioned alloys meet the requirements of both surgeons and patients when they are used for spinal fixation applications. Specifically, surgeons require materials having high Young’s moduli to suppress springback during operations due to the limited space available for operation inside a patient’s body. However, patients require materials with low Young’s moduli for a stress-shielding effect [8, 9]. In other words, the metallic rods used in spinal fixation devices are required to have a low Young’s modulus, good biocompatibility and low springback. Accordingly, it is necessary to develop novel Ti alloys that have good biocompatibility and a changeable Young’s modulus. To accomplish this, it should be possible to change the local Young’s modulus to a high value (>100 GPa) at certain parts of the device by deformation at room temperature, while allowing the Young’s modulus of the rest of the device to remain unchanged at a lower value (close to bone of 10–30 GPa) [10].

Metastable β-type Ti alloys (hereafter β-type Ti alloys) have a low Young’s modulus [11], as well as good mechanical properties and excellent corrosion resistance. Furthermore, the deformation-induced transformation, which can change the Young’s modulus, may occur in β-type titanium alloys; hence, β-type alloys might be the materials of choice for biomedical applications. Additionally, ω phase has a significant effect on the mechanical properties of Ti alloys [12]. The ω phase can be introduced in β-type Ti alloys by deformation-induced phase transformation. However, the deformation-induced phase transformation is dependent on the type of alloy and the stability of the β phase. Hanada and Izumi [13] reported that the ω phase forms during cold working of as-quenched Ti–Cr alloys within a composition range of 8–11.5 mass% Cr. Moreover, the ω phase can be introduced by deformation at room temperature in Ti–V, Ti–Fe and Ti–Mo metastable β-type alloys [14–18]. Cr is known to control the anodic activity of the alloy and increase the tendency of Ti to passivate [19], and the passive films of Ti alloys, in turn, allow them to maintain resistance to corrosion [20–22]. Thus, Cr is suitable as the alloying element to develop Ti-based biomaterials. The Cr content of binary Ti-Cr alloys, however, has to be optimized to produce a deformation-induced ω phase transformation in order to develop a new Ti alloy with a changeable Young’s modulus for spinal fixation applications. The Young’s modulus and tensile properties of the developed alloys have been systematically examined, and the springback and cytotoxicity of the optimized alloys have also been investigated for use in spinal fixation applications.

This review article introduces the topics of research and development of low Young’s modulus β-type titanium alloys with a focus on TNTZ and β-type titanium alloys with changeable Young’s modulus with a focus on Ti–Cr alloys. Shape memory and superelastic titanium alloys as well as surface modification of titanium alloys for desired surface performances were also briefly discussed.

## Low young’s modulus titanium Alloys

### Development of low Young’s modulus titanium alloys

As mentioned previously, when implant devices are implanted to reconstruct disordered bone, prevent bone resorption and enhance good bone remodeling, the Young’s moduli of implants should be close to that of the bone. To satisfy this requirement, many kinds of titanium alloys composed of non-toxic and allergy-free elements exhibiting Young’s moduli closer to that of the bone, i.e., low Young’s modulus titanium alloys, have been developed. Most of them are β-type titanium alloys because their crystal structures are body centered cubic (bcc) where titanium atoms in the β phase are not densely packed like in α- and (α + β)-type titanium alloys where titanium atoms in the α phase having a closed packed (hcp) structure are densely packed [23]. Some of the representative low modulus β-type titanium alloys for biomedical applications are Ti–13Nb–13Zr (ASTM F1713-96), Ti–12Mo–6Zr–2Fe (ASTM F1813-97), Ti–15Mo (ASTM F2066), Ti–16Nb–10Hf, Ti–15Mo–2.8Nb–0.2Si–0.26O, Ti–35Nb–7Zr–5Ta (TNZT), Ti–29Nb–13Ta–4.6Zr (TNTZ), Ti–Mo–Sn, Ti–40Ta and Ti–50Ta [24]. They contain a large amount of high-cost non-toxic and allergy-free elements such as Nb, Ta, Zr and Mo. Among them, TNTZ was developed theoretically by Niinomi et al. [5, 6] using the d-electron alloy design method. TNTZ is composed of Nb, Ta and Zr, which are considered to be the safest alloying elements. Owing to the high cost of rare metals such as Nb, Ta, Mo and Zr, low modulus β-type titanium alloys have very recently been proposed that are based on low-cost elements such as Fe, Cr, Mn, Sn and Al. Examples of such alloys include Ti–10Cr–Al [25], Ti–Mn [26], Ti–Mn–Fe [27], Ti–Mn–Al [28], Ti–Cr–Al [29], Ti–Sn–Cr [30], Ti–Cr–Sn–Zr [31], Ti–(Cr, Mn)–Sn [32] and Ti–12Cr [10].

**Figure 1.**
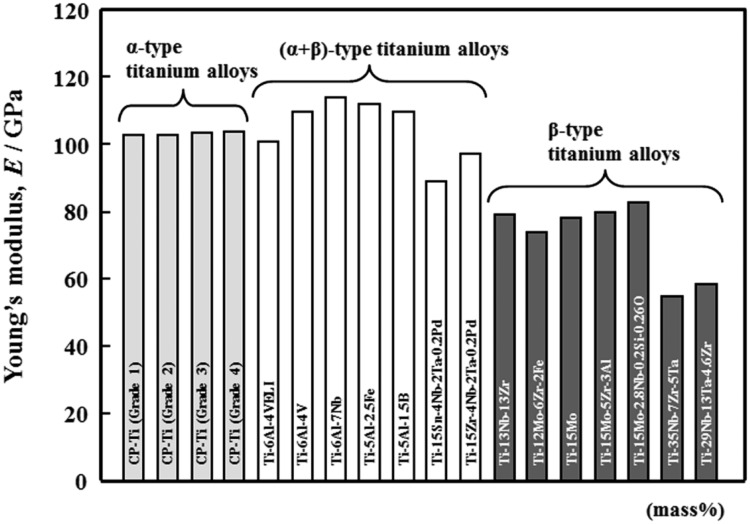
Young’s moduli of representative α-type, (α + β)-type and β-type titanium alloys.

The Young’s moduli of representative low Young’s modulus β-type titanium alloys for biomedical applications are shown in Fig. 1 [33] along with those of the representative α- and (α + β)-type titanium alloys. As mentioned before, the Young’s moduli of the low Young’s modulus β-type titaniuma alloys (generally ∼80 GPa) are much smaller than those of α- and (α + β)-type titanium alloys. The Young’s moduli of TNTZ are listed in Table 1 [34] along with those of Ti64 ELI and bone. TNTZ exhibits the lowest Young’s modulus of ∼55 GPa when subjected to severe cold rolling (CR) after solution treatment (ST) and the highest Young’s modulus of ∼97 GPa when aged after ST. The lowest Young’s modulus of TNTZ is aproximately half of that of Ti64 ELI (110 GPa) but it is still greater than that of the bone (10–30 GPa). Therefore, a further decreas in the Young’s modulus is required for TNTZ. On the other hand, the highest Young’s modulus of TNTZ is close to that of Ti64 ELI. Therefore, from another perspetive, the Young’s modulus of TNTZ can be controlled from the lowest value of ∼55 GPa to the highest value of ∼100 GPa, which is nearly the same as that of Ti64 ELI with both heat treatment and thermomechanical treatment.

**Table 1.** Young’s moduli of (a + b)-type Ti–6Al–4V ELI, β-type Ti–29Nb–13Ta–4.6Zr (TNTZ), and cortical bone

Material	Young’s modulus (GPa)
Ti–6Al–4V ELI (WQ)	110
Ti–29Nb–13Ta–4.6Zr	
WQ	63
WQ + aged at 673 K for 3.6 k	97
WQ + CW	55–60
Cortical bone	10–30

WQ: water quenching after solution treatment, AC: air cooling after solution treatment, CW: severe cold working.

### Enhancing mechanical reliability while maintaining low Young’s modulus

#### Static mechanical reliability

**Figure 2.**
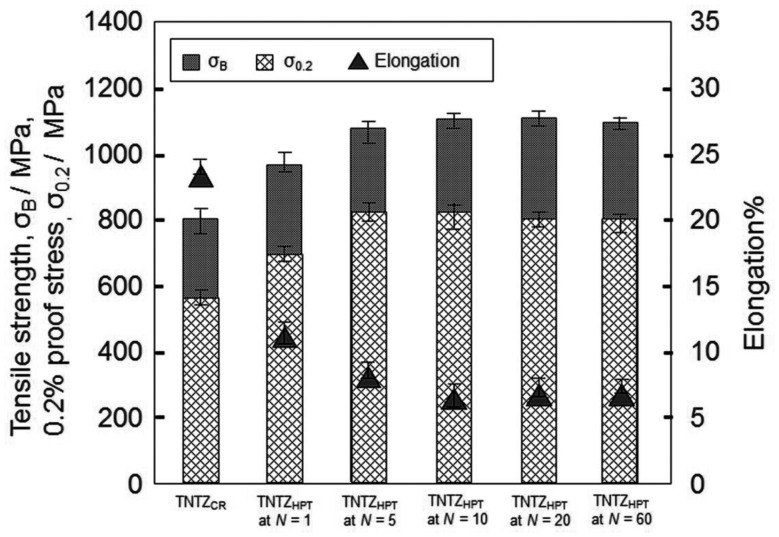
Tensile properties of TNTZ_CR_ and TNTZ_HPT_ at rotation numbers *N = *1–60.

**Figure 3.**
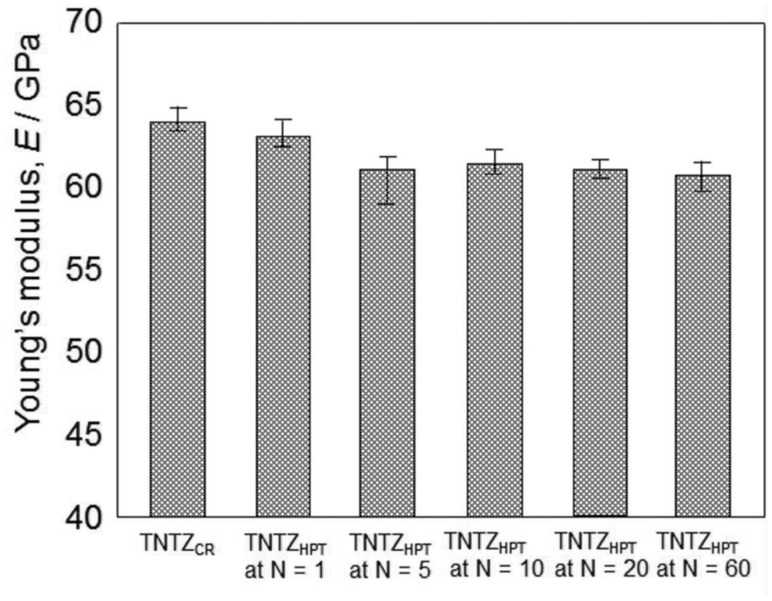
Young’s moduli of TNTZ_CR_ and TNTZ_HPT_ at rotation numbers *N = *1–60.

The lowest Young’s modulus of a low Young’s modulus titanium alloy is, in general, obtained under solutionized conditions leading to poor strength. Therefore, the strength of a low Young’s modulus titanium alloy should be increased while maintaining the low Young’s modulus. The static strength (i.e., tensile strength) of β-type titanium alloys like TNTZ can be improved through severe cold working processes, such as severe CR [12], severe cold swaging [35] and/or severe plastic deformation such as high pressure torsion (HPT) [36, 37]. Such processes have the potential to increase tensile strength to the levels similar to or greater than that of Ti64 ELI, while still retaining good ductility (elongation), as the high degree of dislocation introduced creates a great deal of work hardening. Figure 2 [37] shows an example of the tensile properties of TNTZ subjected to severe CR after ST (TNTZ_CR_) and TNTZ subjected to HPT at a rotation number *N* = 1–60 (TNTZ_HPT_ at *N* = 1–60). The tensile strength, 0.2% proof stress, and elongation of TNTZ_CR_ are 800 MPa, 565 MPa and 22.5% on average, respectively. The corresponding values of TNTZ subjected to ST have been reported to be ∼600 MPa, 370 MPa and 26%, respectively. The tensile strength of TNTZ is increased by severe CR. It is clearly seen that the tensile strength and the 0.2% proof stress of TNTZ_HPT_ are greater than those of TNTZ_CR_. Further, the elongation of TNTZ_HPT_ shows a reverse trend, i.e., it is less than that of TNTZ_CR_. Figure 3 [37] shows the Young’s moduli (*E*) calculated from a stress–strain curve obtained by tensile tests for TNTZ_CR_ and TNTZ_HPT_ at all *N.* After inducing severe torsional strain, the Young’s modulus of TNTZ_HPT_ slightly declines at *N* < 5 and then tends to saturate at *N* ≥ 5 with increasing *N*. The Young’s modulus of TNTZ_HPT_ decreases from 64 GPa for TNTZ_CR_ to ∼60 GPa for TNTZ_HPT_ at *N* = 60, which represents a decline of 6%. However, it is important to note that the Young’s modulus of TNTZ_HPT_ tends to become saturated at *N* > 5.

**Figure 4.**
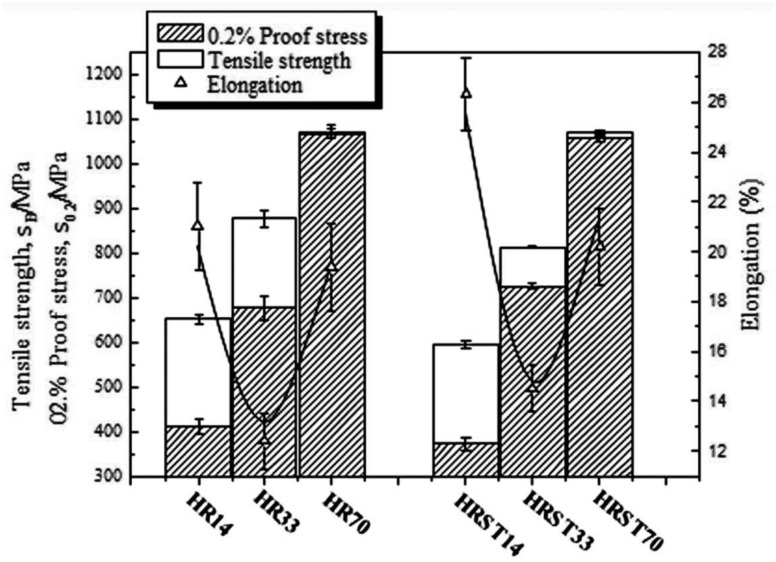
Tensile properties: tensile strength, 0.2% proof stress and elongation of TNTZ-0.14O, TNTZ-0.33O and TNTZ-0.70O subjected to hot rolling (HR14, HR33 and HR70), and solution treatment at 1003, 1083 and 1243 K for 3.6 ks after hot rolling (HRST14, HRST33 and HRST70).

**Figure 5.**
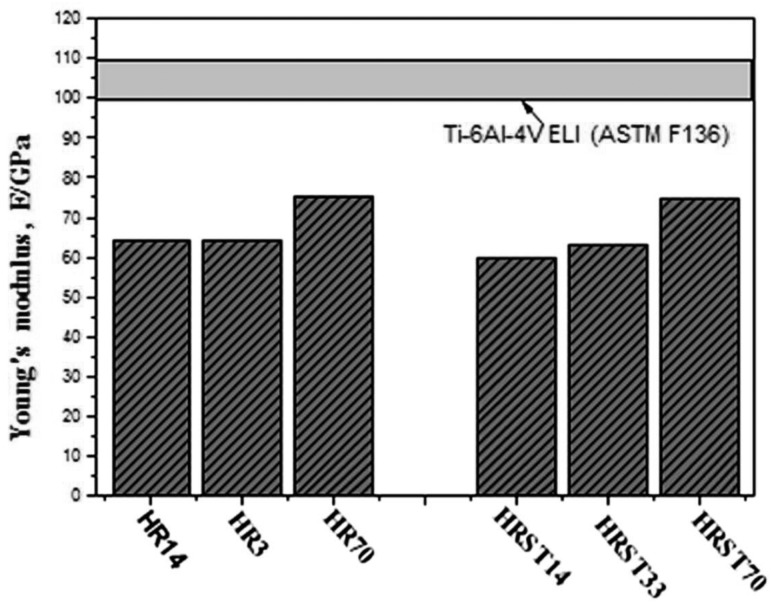
Young’s moduli of TNTZ-0.14O, TNTZ-0.33O and TNTZ-0.70O subjected to hot rolling (HR14, HR33 and HR70), and solution treatment at 1003, 1083 and 1243 K for 3.6 ks after hot rolling (HRST14, HRST33 and HRST70), respectively.

Solid solution strengthening by oxygen (O) is also effective in improving the strength of TNTZ while maintaining a low Young’s modulus. Figure 4 [38] shows the tensile strength, 0.2% proof stress and elongation of TNTZ with oxygen content of 0.14 (TNTZ-0.14), 0.33 (TNTZ-0.33) and 0.70 (TNTZ-0.70) in mass% subjected to hot rolling designated as HR14, HR33 and HR70, and subjected to ST after hot rolling, designated as HRST14, HRST33 and HRST70. With an increase in the oxygen content, the tensile strength and 0.2% proof stress of all TNTZ variants increase, but their elongation firs decreases and then increases. This result is contradictory to that reported conventionally. Their tensile strength can reach ∼1100 MPa, and their elongation can reach ∼20% of those of HR70 and HRST70. Both the tensile strength and the elongation of HR70 and HRST70 are larger than those of Ti64 ELI registered in the ASTM F136 standard [1]. Figure 5 [38] shows the Young’s moduli of HR14, HR33, HR70, HRST14, HRST33 and HRST70 along with that of Ti64 ELI registered in the ASTMF136 standard [1]. The Young’s moduli of TNTZ after both hot rolling and ST increase with increasing oxygen content. The Young’s moduli of HR14, HR33, HRST14 and HRST33 are <65 GPa, and those of HR70 and HRST70 are less than ∼75 GPa, which is much less than that of Ti64 ELI (∼100–110 GPa). The microstructure and X-ray diffraction profiles of HR14, HR33, HR70, HRST14, HRST33 and HRST70 revealed the presence of a single phase. Therefore, the increase in the Young’s moduli of TNTZ after hot rolling and ST can be attributed to oxygen dissolution in the phase.

#### Dynamic mechanical reliability

It is important to note that the dynamic strength (i.e., fatigue strength) is not improved by either severe cold working or severe plastic deformation [12]. Consequently, the most effective means of improving the fatigue strength of β-type titanium alloys is the introduction of a secondary phase, or secondary particles, into the β-phase matrix, through either aging or direct addition of particles of hard materials such as ceramics.

It is known that ω-phase precipitation significantly increases the strength and Young’s modulus compared with α-phase precipitation although ω-phase also enhances the brittleness of the alloy. A small amount of ω-phase precipitation is therefore expected to improve the fatigue strength of TNTZ, while maintaining a low Young’s modulus. For this purpose, a short-aging time at fairly low temperatures is effective in producing a small amount of ω-phase precipitation [39]. The Young’s moduli of TNTZ subjected to ST, severe CR and aging after CR at 573 K can approach a value of <80 GPa, which is a tentative target value for a low Young’s modulus alloy for biomedical applications. In this case, the fatigue strength of TNTZ can be seen to improve by aging treatment for 10.8 ks to a fatigue limit of ∼600 MPa when compared with that of TNTZ subjected to ST showing a fatigue limit of 350–400 MPa, while maintaining the Young’s modulus at <80 GPa. A transmission electron microscopic micrograph confirmed the distribution of ω-phase, thus demonstrating that it is possible to effectively utilize the controlled precipitation of ω-phase to improve the fatigue strength of TNTZ, while maintaining a low Young’s modulus.

The addition of a small amount of ceramic particles such as TiB_2_ or Y_2_O_3_ into the matrix is also expected to improve the fatigue strength of β-type titanium alloys, while maintaining a low Young’s modulus. This was confirmed by severe CR of TNTZ containing TiB_2_ or Y_2_O_3_ [40, 41], where Y or B contents are 0.1 and 0.2 mass% for B, or 0.05 and 0.1 mass% for Y, respectively, showing an improved fatigue strength of 550–600 MPa or 500–550 MPa, while their Young’s moduli are ∼60 MPa.

The fatigue strength of TNTZ can also be enhanced by solid solution strengthening by O solute [42]. The fatigue strength of TNTZ with an O content of ∼0.5 mass% exhibits a fatigue limit of ∼600 MPa with a Young’s modulus of ∼70 GPa.

### Biological and mechanical biocompatibility

Biological biocompatibility refers to chemical reactions with living tissue, and is expressed by indications such as toxicity, allergy and fusing between biomaterials and living tissue. Mechanical biocompatibility refers to the mechanical suitability of biomaterials as living tissue and is expressed by the suitable level of Young’s modulus as well as tensile strength, ductility, fatigue life, fretting fatigue life, wear properties, functionalities, etc., in a broad sense [24]. Among these mechanical biocompatibility indicators, the Young’s modulus is one of the most significant. To investigate biological biocompatibility and mechanical biocompatibility, indicators such as Young’s modulus and animal studies are highly required.

**Figure 6.**
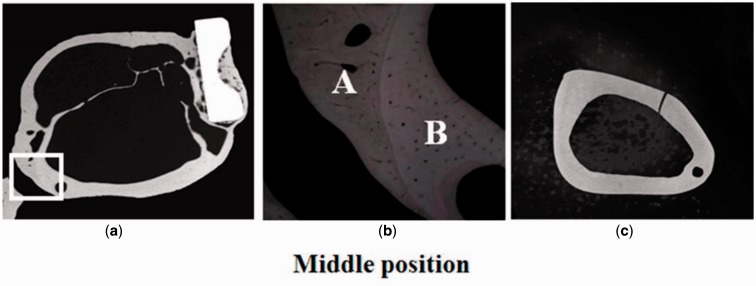
CMRs (Carbon-13 Nuclear Magnetic Resonance) of cross sections of fracture models implanted with and without bone plates made of TNTZ at middle position at 48 weeks after implantation: (**a**) cross section of fracture model, (**b**) enlarged view of the selected area in (a), namely high-magnification CMR of branched parts of bones formed outer and inner sides of tibiae, and (**c**) cross sections of unimplanted tibiae.

Studies on Japanese white rabbits have been conducted using TNTZ, SUS 316 L stainless steel, and Ti64 ELI to investigate the effects of intramedullary rod implantation [6] and bone plate implantation [8]. The Young’s moduli of the intramedullary rods of TNTZ, Ti64 ELI and SUS 316 L stainless steel in these cases were measured using three-point bend testing to be 58, 108 and 161 GPa, respectively [6]. For the implantation of intramedullary rods and bone plates, both the lowest bone atrophy and best bone remodeling were reported for TNTZ. In the study on bone remodeling, utilizing bone plates made of TNTZ, Ti64 ELI and SUS 316 stainless steel implanted in fracture models made in the rabbit tibiae, the healing conditions were observed using X-ray photographs taken at regular intervals over a period of up to 48 weeks after implantation. Following this, both tibiae were extracted along with the bone plate, and the bone formation was externally observed. For all the materials, bone union was obtained at 4 weeks after implantation (4w), and the fracture line was barely discernible at 8 weeks after implantation (8w). Moreover, the trace of the experimental fracture was completely absent at 16–20 weeks after implantation (16w and 20w, respectively). However, bone atrophy (thinning of the bone) was observed under the bone plate, and the time at which this occurred varied between different materials. According to the X-ray images taken from 4w after implantation to 18 weeks after implantation (18w) for each plate, in SUS316 stainless steel, the thinning of the bone was first observed at 7 weeks after implantation, and the bone almost disappeared at 12 weeks after implantation (12w). In Ti64 ELI, the thinning was first observed at 7 weeks after implantation, and almost disappeared at 14 weeks after implantation (14w). Finally, in TNTZ, the corresponding time was 10 weeks and 18 weeks after implantation. Furthermore, an increase in the diameter of the tibia and in the double wall structure in the intramedullary bone tissue was observed only in the case of the bone plate made of TNTZ, as shown in Fig. 6 [8]. In this figure, the inner wall bone structure represents the original bone, i.e., the remaining old bone, whereas the outer wall bone structure is the newly formed part. This bone remodeling is the direct result of using a bone plate with a low Young’s modulus. The low Young’s modulus is found to be effective in preventing bone resorption and leading to excellent bone remodeling.

**Figure 7.**
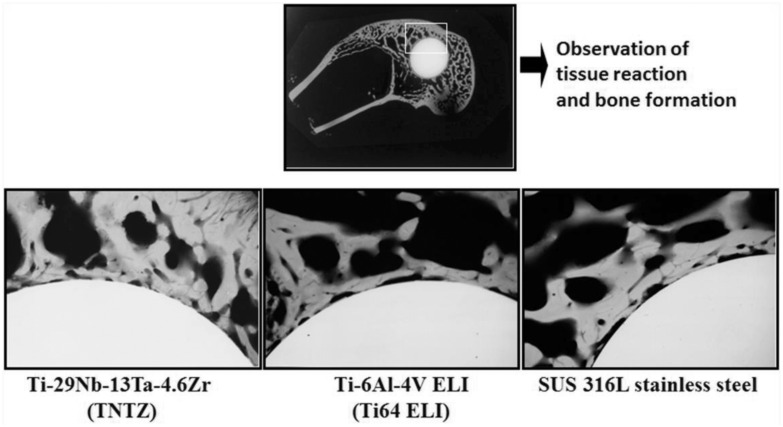
CMR. Photograph of boundary of each specimen and bone at 8 weeks after implantation.

To investigate the biological biocompatibility of TNTZ, small round bars made of TNTZ, Ti64 ELI or SUS 316 L stainless steel were implanted in lateral femoral condyles of the rabbit. Then, a contact micro radiogram (C.M.R) of the boundary of bone and each implant was examined, and the results are shown in Fig. 7 [6]. Each specimen is surrounded by newly formed bone, and the bone tissue shows partial direct contact with the specimen. However, the extent of the direct contact is greater with TNTZ than with Ti64 ELI and SUS 316 L stainless steel. The biological biocompatibility of TNTZ with bone is excellent.

## Titanium alloys with changeable young’s modulus and low young’s modulus

### Development of titanium alloys with changeable young’s modulus and low young’s modulus

**Figure 8.**
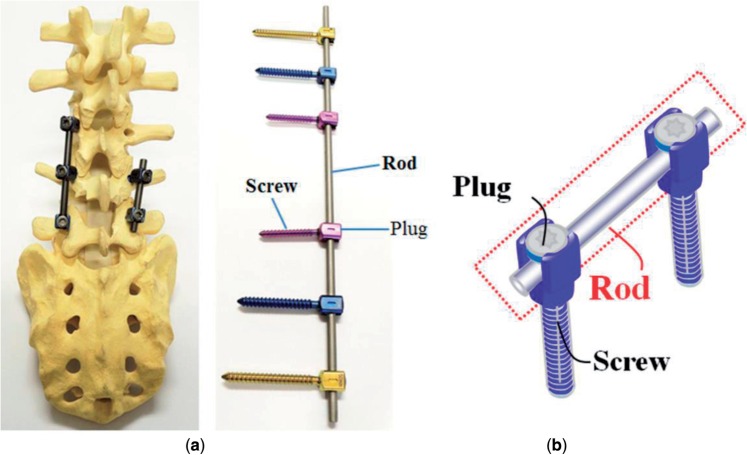
(**a**) Images and (**b**) schematic drawing of spinal fixation system consisting of rods, screws and plugs.

**Figure 9.**
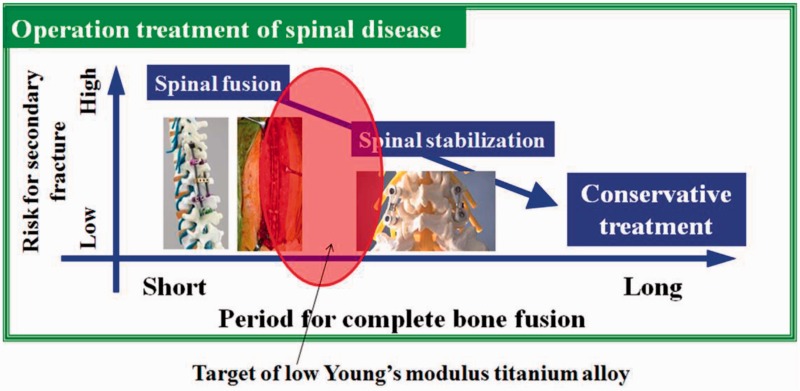
Schematic drawing of risk for secondary fracture and period for complete bone fusion in operation treatment of spinal disease.

**Figure 10.**
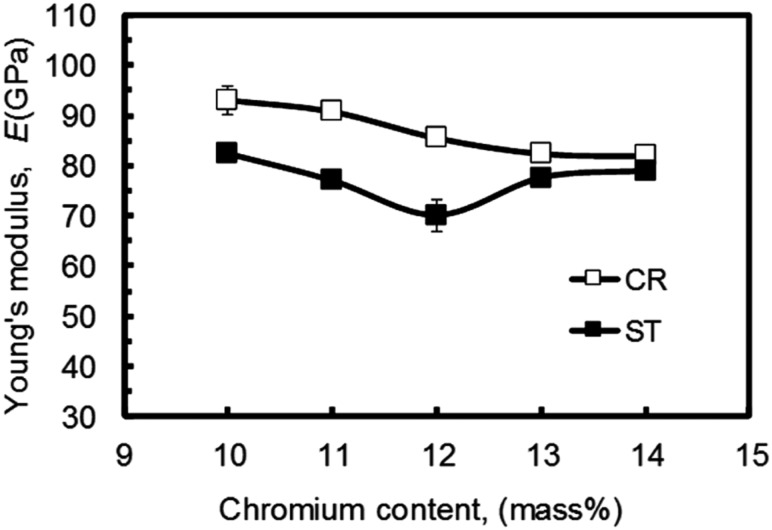
Young’s modulus of Ti–(10-14)Cr alloys subjected to solution treatment and cold rolling.

**Figure 11.**
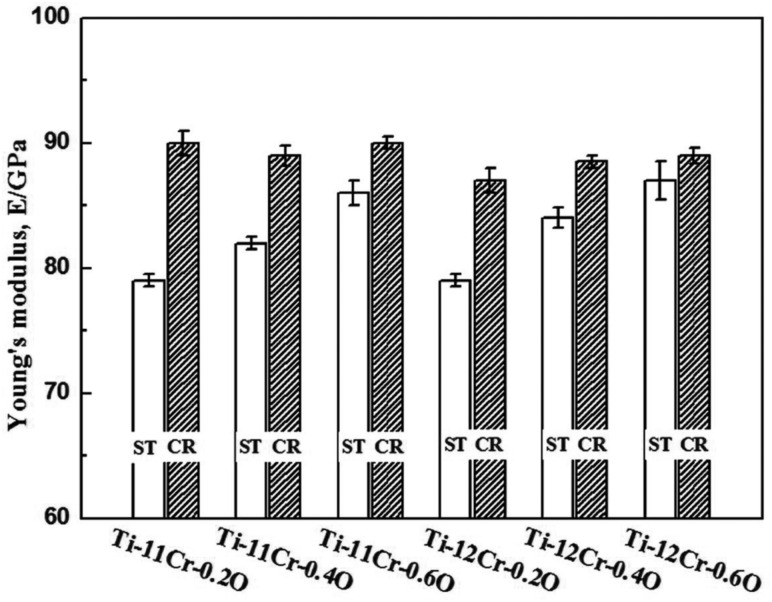
Young’s moduli of Ti-(11,12) Cr-(0.2,0.6) O alloys subjected to solution treatment (ST) and 10% reduction cold rolling (CR).

Taking the spinal fixation devices, which are generally composed of rods, plugs and screws as schematically shown in Fig. 8 [43], as an example, the rods, in particular, undergo bending when manually handled by surgeons within the small space inside the patients’ body for in situ spine contouring [10]. The bending shape should be maintained. Therefore, bend back, called springback, of the bent rod should be prevented. The amount of springback in implant rods should be small so that the implant offers better handling ability during operations. The amount of springback is considered to depend on both the strength and the Young's modulus of the implant rod. If two implant rods having the same strength, but different Young's moduli are used, the implant rod having the lower Young's modulus shows greater springback, as schematically shown in Fig. 9 [43]. Therefore, patients require low Young’s modulus to prevent stress shielding, whereas surgeons require high Young’s modulus to prevent springback. To satisfy these conflicting demands at the same time, it should be possible to change the Young’s modulus to a high value only at the bent parts of the rod by deformation at room temperature, while allowing the Young’s modulus of the rest of the rod to remain unchanged at a low value [10]. Among these non-equilibrium phases such as α′ martensite, α″ martensite, and ω-phase, the ω phase has a higher Young’s modulus than the β phase. Therefore, if ω phase is induced by deformation (bending) at only the deformed part, the conflicting demands might be satisfied. For this purpose, Ti-12Cr has been developed. Fig. 10 [44] shows the Young’s moduli of Ti–(10-14)Cr alloys subjected to ST and CR, where CR at a reduction ratio of 10% was performed to simulate deformation. The degree of increase in Young’s modulus is the highest at a Cr content of 12 mass%. To achieve a higher degree than that of Ti–12Cr, which has an athermal ω-phase, O is added to the alloy because O suppresses the formation of athermal ω-phase. Finally, Ti–11Cr–0.2O, which exhibits a higher degree of increase in Young’s modulus than that of Ti-12Cr, has been developed as shown in Fig. 11 [44].

### Trend of springback

**Figure 12.**
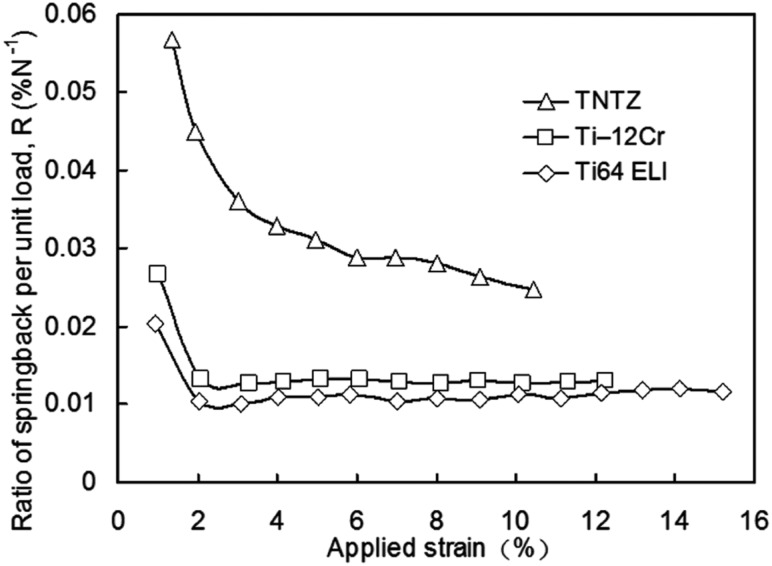
Ratio of springback per unit load as a function of applied strain for Ti–12Cr, Ti64 ELI, and TNTZ, and strains for calculation of the springback ratio.

Figure 12 [45] shows comparison profiles of the ratio of springback per unit stress as a function of the applied strain for TNTZ, Ti-12Cr and Ti64 ELI (Ti64 ELI). The ratios of springback per unit stress of all the alloys show a similar trend, i.e., the value initially decreases significantly and then remains approximately stable with increasing applied strain. Among the mentioned alloys, Ti–11Cr–0.2O, which reaches a very low yet stable value when the applied strain is >2%, exhibits a minimal ratio of springback per unit stress. This value is much lower than that of TNTZ and is the closest to that of Ti64 ELI among the compared alloys.

### Cytotoxicity

**Figure 13.**
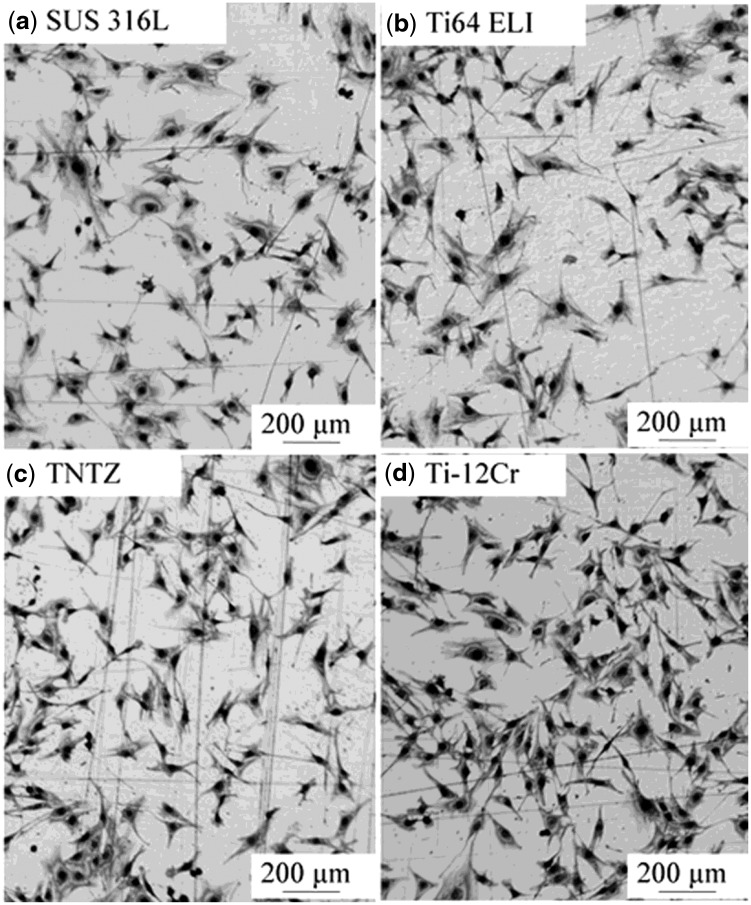
Optical images of MC3T3-E1 cells cultured in Ti–12Cr alloy and the alloys considered for comparison for 24 h.

**Figure 14.**
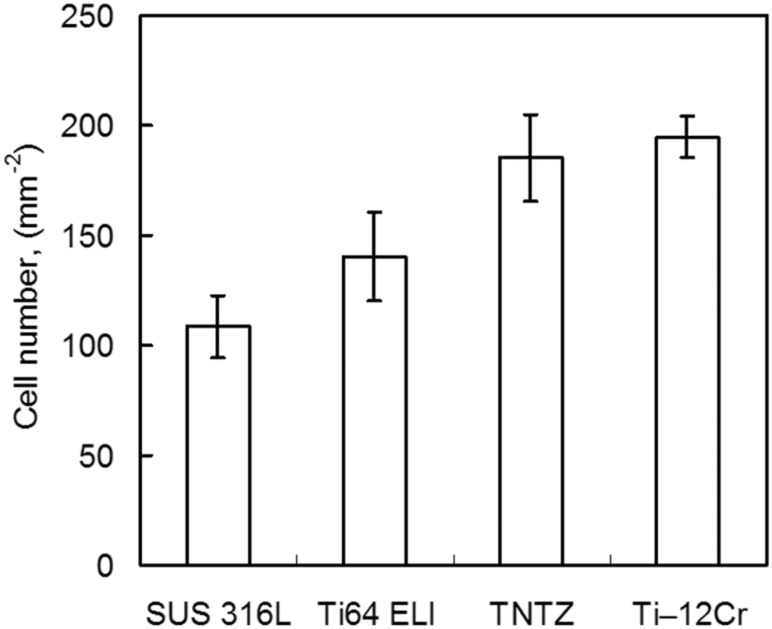
Density (cell number) of MC3T3-E1 cells cultured in Ti–12Cr alloy and the alloys considered for comparison for 24 h.

Figure 13 [44] shows the morphologies of MC3T3-E1 cells cultured for 86.4 ks on Ti-12Cr and the alloys considered for comparison. All alloys show healthy morphologies of cells with a flattened spindle shape. Figure 14 [44] shows the density of cells cultured for 86.4 ks on Ti-12Cr and the alloys considered for comparison. Ti–12Cr has the highest cell density, which is considerably higher than that of SUS 316 L and Ti64 ELI, and similar to that of TNTZ. These findings indicate that Cr in Ti–12Cr can increase the tendency of Ti to passivate [46]. The passive film of Ti–12Cr allows it to maintain high resistance to corrosion and prevent release of Cr ions from Ti–12Cr. Therefore, the excellent cell density may be attributed to the passive film formed on the surface of Ti-12Cr.

**Figure 15.**
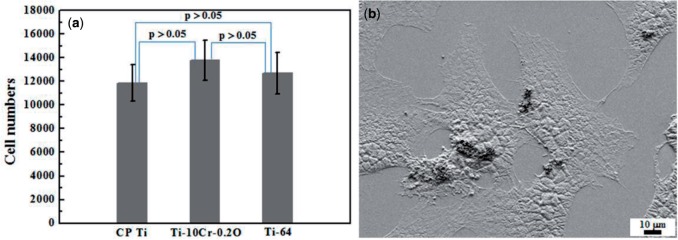
Cytocompatibility of the alloys. (**a**) Cell numbers after culturing for 7 days on CP ti, Ti–10Cr–0.2O, and Ti64 ELI (Ti-64) and (**b**) SEM image of cells cultured on Ti–10Cr–0.2O.

Figure 15a [47] shows the osteoblast cell numbers after a culture time of 6 days on CP Ti, Ti64 ELI, Ti–10Cr–0.2O-ST (Ti–10Cr–0.2O subjected to ST), and Ti–10Cr–0.2O-CR (Ti–10Cr–0.2O subjected to CR at a reduction ratio of 10%). Ti–10Cr–0.2O-ST and Ti–10Cr–0.2O-CR show comparable cell numbers after 6-day culturing in comparison with CP Ti and Ti64. Figure 15b [47] shows a scanning electron microscope image displaying the morphology of the cells cultured on Ti–10Cr–0.2O-ST. The cells exhibit a flattened cellular morphology with a high level of attachment, which indicates that the cells can spread well on the surface of Ti–10Cr–0.2O-ST components.

Therefore, Ti–Cr alloys exhibit good cell biocompatibility and are expected to show good living tissue compatibility.

## Applicability of titanium alloys with changeable young’s modulus to rods of spinal fixation devices

**Figure 16.**
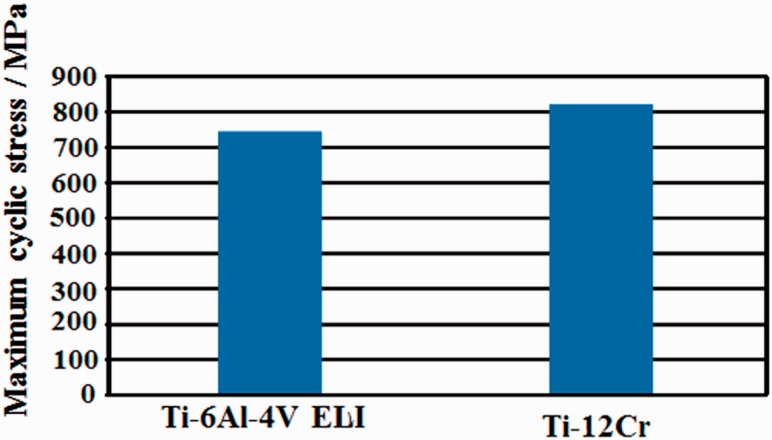
Fatigue limit of Ti–12Cr and Ti–6Al–4V ELI.

**Figure 17.**
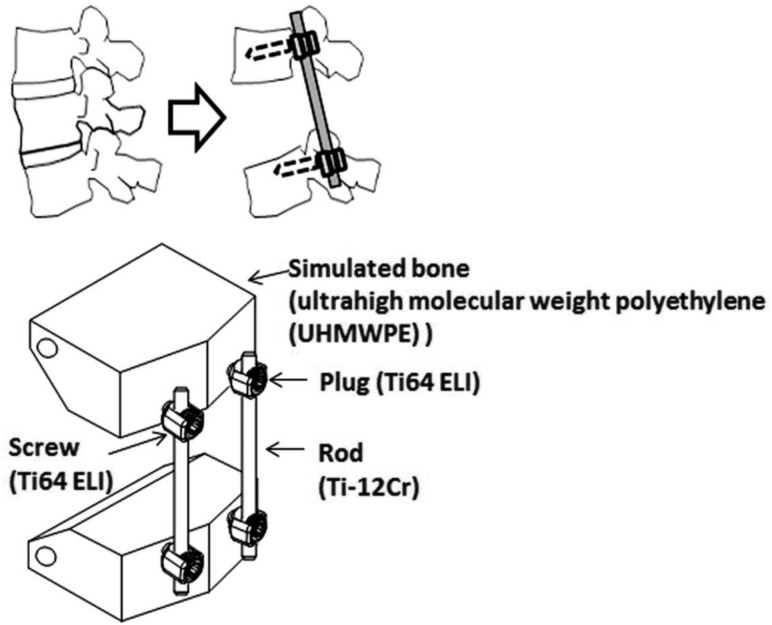
Schematic drawing of compressive fatigue strength test method according to ASTM F1717.

**Figure 18.**
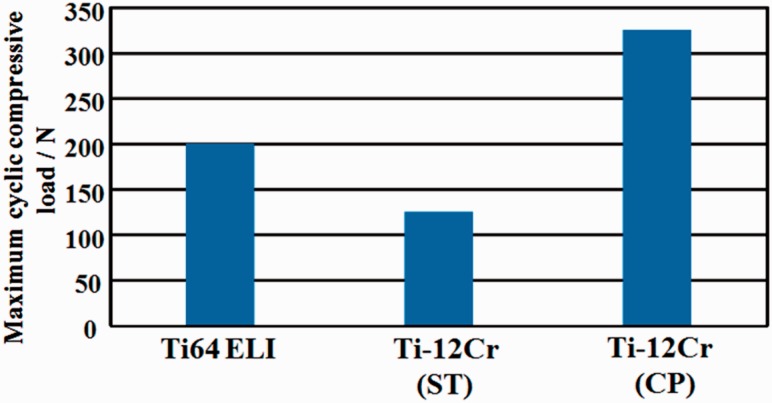
Compressive fatigue limit of Ti–6Al–4V ELI (Ti64 ELI), and Ti–12Cr subjected to ST and cavitation peening after solution treatment (CP) evaluated according to ASTM F 1717.

**Figure 19.**
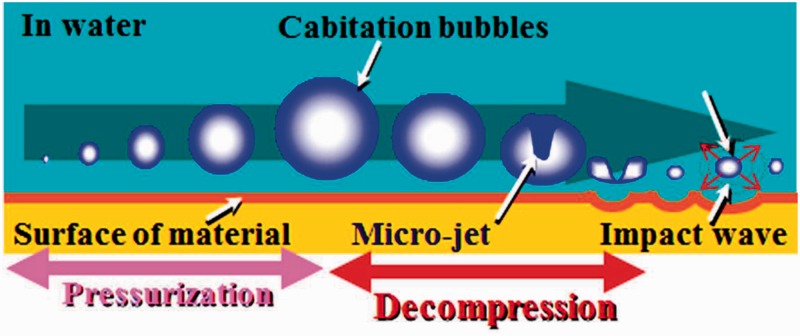
Schematic drawings of development and crashing of cavitation.

In the application of titanium alloys with changeable Young’s modulus for the rods of spinal fixation devices, their fatigue strength is highly important factors. The uniaxial fatigue strength of titanium alloy with changeable Young’s modulus, Ti–12Cr, is significantly excellent as shown in Fig. 16 [48], which shows the fatigue limits of Ti–12Cr and Ti64 ELI for comparison. The fatigue ratio, which is a ratio of fatigue limit to tensile strength, of Ti-12Cr is ∼0.9, whereas that of Ti64 ELI is ∼0.6. However, to use the alloy in practical applications for rods, its endurance must be evaluated in a laboratory according to ASTM F1717 [49], which describes a testing method used for evaluating the compressive fatigue strength of spinal fixation rods via a simulated spinal fixation model as shown in Fig. 17 [49]. The spinal fixation device comprises screw, and plug made of Ti64 ELI and rod made of Ti–12Cr. A Ti64 ELI rod is also used for comparison. Bone is simulated using ultrahigh molecular weight polyethylene (UHMWPE). The compressive fatigue limit of Ti–12Cr subjected to ST is less than that of Ti64 ELI, as shown in Fig. 18 [50]. In the ASTM F1717 compressive fatigue testing, the rod typically fails at the contact area between the rod and the plug. Therefore, fretting that occurs between the rod and the plug is thought to reduce the compressive fatigue strength of the rod. An effective solution to such a problem is improving the mechanical properties and tribiological characteristics of the rod. The introduction of a hardened layer via compressive residual stress on the surface of the rod effectively prevents fretting fatigue. Peening techniques can introduce these hardened layers through plastic deformation, i.e., work hardening, by delivering a large impact on the material surface. Among the major peening techniques, cavitation peening, which is schematically shown in Fig. 19 [50], appears to be a highly promising method for improving the compressive fatigue strength of rods used in spinal fixation devices because it induces less surface damage than other peening techniques. Therefore, cavitation peening was performed on Ti–12Cr rods to improve their compression fatigue strength as evaluated by ASTM F1717; the technique significantly increases the compressive fatigue strength of the rods, as also shown in Fig. 18 [50].

Therefore, Ti–12Cr with changeable Young’s modulus is expected to be used in practical applications for the rods of spinal fixation devices.

## Applicability of shape memory and superelastic titanium alloys to rods of spinal correction

It is usually essential that biomedical shape memory titanium alloys should fulfill the requirements of good biocompatibility, superior corrosion resistance and stable shape memory property. To date, only Ti–Ni alloys meet the requirements and have therefore been largely commercialized for biomedical applications [51]. Shape memory and superelastic titanium alloys have extensive orthopedic applications, e.g., spinal correction. Through heating, ‘self correct’ can be accomplished when the shape memory rod resumes to its memory shape. The force generated by this process accelerates healing, reducing the time of recovery. Nitinol shape memory alloy (SMA) rod-based correction by comparing the clinical and radiographic results can be obtained from using a temporary SMA rod and those from a standard rod in the correction of severe scoliosis. The temporary use of SMA rod is reported to be able to reduce operative time, blood loss and achieve better coronal correction rates, while improve the correction of the coronal plane when compared with standard techniques [52]. However, the Ni-hypersensitivity and toxicity of Ni have stimulated the development of Ni-free shape memory alloys. Nowadays it seems that β-Ti alloys are the most attractive candidates for biomedical shape memory and superelastic alloys. Ti–Nb–X (X = Zr, Ta, Mo, Au, Pd, Pt, Al, Ga, Ge and O) and Ti–Mo–X (X = Ta, Nb, Zr, Au, Pd, Pt, Al, Ga and Ge) alloys have been developed, and their shape memory effect and superelasticity were investigated systematically in the past decade [53–55]. Yet extensive efforts are still needed for the alloys for potential clinical applications.

## Surface physicochemical modification of titanium and titanium alloys

**Figure 20.**
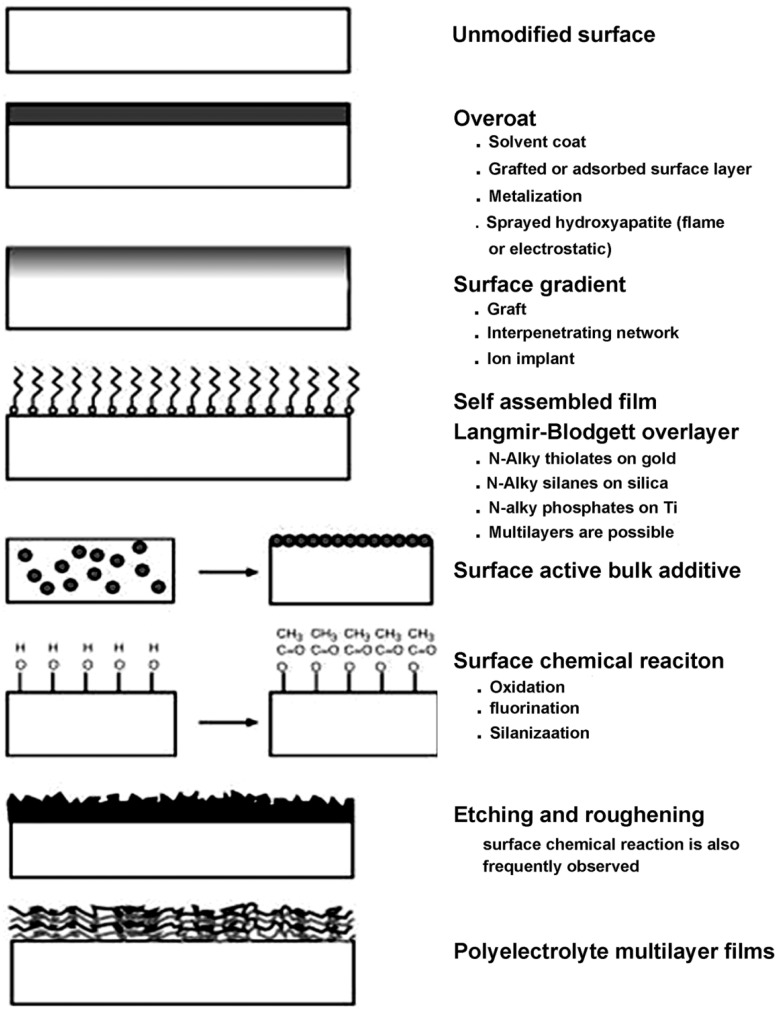
Schematic representation of the surface modification approaches.

The aforementioned bulk properties of titanium and its alloys have been recognized to be highly relevant in terms of element design and fabrication processing. It is established that the biological response to biomaterials and devices is controlled largely by physicochemical characteristics of their surfaces. Wear resistance, corrosion resistance, lubricity, blood compatibility, cell adhesion and growth, protein adsorption, transport properties and electrical characteristics of biomaterials can be selectively improved or changed using physicochemical surface treatment techniques while retaining the bulk properties and modifying only the outermost surface [56]. In most cases, surface modifications of the titanium alloys are necessarily required for biomedical applications. According to the formation style, surface modifications basically fall into two categories: (i) chemically or physically altering the atoms, compounds, or molecules in the existing surface (chemical modification, etching, mechanically roughening) or (ii) overcoating the existing surface with a material having a different composition (coating, grafting and thin film deposition) (Fig. 20) [][56]. Various surface modification methods [57], e.g., thermal spraying, sol–gel, chemical and electrochemical treatment, ion implantation and mechanical methods, etc., have been developed in response to the deficient biocompatibility or mechanical strength of titanium and titanium alloys which no longer function properly due to degradation from wear or tendency to release metallic ions attributing to potential corrosion in the biological environments. For hard-tissue replacement, the titanium alloy implants are usually coated by more biocompatible materials, e.g., calcium phosphate bioceramics. Owing to its similarity in chemistry to natural bone mineral, calcium phosphate in particular hydroxyapatite has been extensively used for making surface coatings for orthopedic applications [58–60]. Among the surface coating techniques that have been attempted for making the biomedical coatings, thermal spray in particular plasma spray is the most widely used approach, which is so far the only certified method by Food and Drug Administration for applying CaP coatings to prosthesis surfaces [58]. From the perspective of long-term performances of the implant, satisfying bonding strength and high crystallinity in plasma sprayed CaP coatings are considered desirable. With the development of the surface engineering, some new techniques, e.g., low-pressure plasma spraying [61], solution precursor plasma spraying [62], cold spray [63] and aerosol deposition [64], have been attracting increasing attentions because they offer new opportunities of making unique and distinctive coatings for biomedical applications. In addition, a serious issue related to titanium-implanted devices is the bacterial infection, which is one of the biggest complications following surgery. To prevent such infections, some approaches have been applied to improve the antibacterial ability of the materials. Exciting progresses have been made in recent years; however, antibacterial coatings on titanium alloy [65], titanium embedded with biocidal agent [66], etc., are still not widely used clinically. The development of novel titanium alloys, however, brings new challenges and chances for the existing surface modification techniques.

## Summary

Low Young’s modulus titanium alloys composed of non-toxic and allergy-free elements are highly expected to be used in practical applications for load bearing-implants such as stems of artificial hip joints, bone plates, spinal fixation devices, artificial dental roots, etc., because they are effective in preventing stress shielding, which leads to bone resorption and poor bone remodeling. When springback should be prevented to keep the deformed shape of implants, e.g., in the case of the rod of a spinal fixation device, titanium alloys with changeable Young’s modulus are expected to be advantageous. In the case of spinal fixation devices, these titanium alloys are expected to reduce adjacent segmental diseases after spinal fusion because the rods made of them are flexible. However, the efficiency of the low Young’s modulus must be proven to obtain approval. There are three treatments for spinal fixation, namely spinal fusion, spinal stabilization and conservative treatment. When low Young’s modulus titanium alloys are used for the rods of spinal fixation devices, the target is expected to be between spinal fusion and stabilization. To attain extensive biomedical applications of the titanium alloys, surface modification is usually expected. It is anticipated that novel surface modification techniques could open doors for the low Young’s modulus titanium alloys for appropriate properties for biomedical applications. [][]
